# Polymer Membrane Modified with Photocatalytic and Plasmonic Nanoparticles for Self-Cleaning Filters

**DOI:** 10.3390/polym15030726

**Published:** 2023-01-31

**Authors:** Aliaksandr Burko, Siarhei Zavatski, Arina Baturova, Makhina Kholiboeva, Julia Kozina, Kseniya Kravtsunova, Vladimir Popov, Artem Gudok, Sergey Dubkov, Stanislav Khartov, Hanna Bandarenka

**Affiliations:** 1 Applied Plasmonic Laboratory, Belarusian State University of Informatics and Radioelectronics, 220013 Minsk, Belarus; 2 Institute of Advanced Materials and Technologies, National Research University of Electronic Technology, Moscow 124498, Russia; 3 Department of General and Applied Physics, Moscow Institute of Physics and Technology, Moscow 141701, Russia; 4 Federal Research Center «Krasnoyarsk Scientific Center», Siberian Branch, Russian Academy of Sciences, Moscow 119991, Russia

**Keywords:** polymer membrane, titanium dioxide nanoparticles, photocatalysis, plasmonic silver nanoparticles, permeability, SERS-detection

## Abstract

In this study, we developed a filtering material for facial masks, which is capable of trapping and subsequent inactivation of bacteria under white light emitting diodes (LED) or sunlight irradiation. Such a functionality is achieved via the modification of the composite membrane based on porous polymer with photocatalytic (TiO_2_) and plasmonic (Ag) nanoparticles. The porous polymer is produced by means of a computer numerical control machine, which rolls a photoresist/thermoplastic mixture into a ~20-µm-thick membrane followed by its thermal/ultraviolet (UV) hardening and porosification. TiO_2_ nanoparticles are prepared by hydrothermal and sol-gel techniques. Colloidal synthesis is utilized to fabricate Ag nanoparticles. The TiO_2_ photocatalytic activity under UV excitation as well as a photothermal effect generated by plasmonic Ag nanoparticles subjected to LED irradiation are studied by the assessment of methylene blue (MB) decomposition. We demonstrate that, in contrast to the filter of the standard facial medical mask, the polymer membrane modified with spray-coated TiO_2_ and Ag nanoparticles prevents the penetration of *bacillus subtilis* from its top to bottom side and significantly inhibits bacterial growth when exposed to LED or sunlight.

## 1. Introduction

Currently, the main approach in the manufacture of the most affordable facial masks designed to protect the human respiratory organs from bacteria and viruses is to sew them using three components: the outer coating, the filter pad, and the material touching the user’s skin. The composition of such masks hardly guarantees an effective prevention of infection penetration to the user’s respiratory system. At the same time, a lot of people do not have the opportunity to use alternative personal protective equipment because of its expensiveness, with open purchasing only in wholesale batches, and absence on the market during high demand periods due to the long and complicated manufacturing process. Existing air-filtering materials exploit the size exclusion principle to cut the primary route of microbes’ invading the human body. This means that geometrical parameters of pores in the filtering material dictate what particles will be stopped on the way to breathing organs. New findings, however, indicate that having the filters acting exclusively as a physical barrier is not a sustainable solution for contagious diseases because, after the contact with pathogens, the mask turns into a source of further infection that could be spread by simple touching if not handled properly [1,2]. This will very likely happen if millions of untrained people use such a protective measure. The other issue of existing medical masks is their uselessness for capturing many microorganisms (e.g., viruses) with a rather small diameter that varies from 100 to 160 nm [3]. The manufacture of flexible filtering materials with such pore sizes is still a non-trivial task. Prospective candidates for such filters are porous membranes made of polymers atypical for the medical field (e.g., epoxy resins or photoresists), which can be easily formed using mastered techniques, e.g., the Dr. Blade method [4] or a spin-coating [5], for producing films of well-defined thickness followed by lifting the coating up from the substrate. A porosification of the polymer film in this case can be derived by traditional UV lithography [6], interference lithography [7], or removal of a sacrificial component from the polymer-based blend [8], to name a few.

A challenge in producing advanced facial masks is imparting a self-cleaning property to the filtering material, which is possessed under an excitation of the optical range from widely available sources, such as the sun or/and daylight lamps. The latter are often used in crop production [9] or animal husbandry [10]. Therefore, the self-disinfecting respiratory filters for employees, working under such conditions, are of a great need because of the frequent mutation and transmission of animal viruses to humans who are in direct contact with them. The latest advances in the design and engineering of nanostructures possessing photocatalytic activity should help to solve this problem. In particular, titanium dioxide is one of the most popular photocatalysts that has already proved its prospects for photosynthesis applications and photoreduction of carbon dioxide [11,12,13,14]. Titanium dioxide (TiO_2_) nanoparticles have often been utilized to provide the destruction of pathogens due to their photocatalytic activity when exposed to electromagnetic radiation [15,16,17]. In most cases, studies of TiO_2_ nanoparticles have been focused on the development of water disinfection methods [18,19]. However, several studies have reported a use of the TiO_2_ nanoparticles for the destruction of malignant cells in the human body [20,21]. Conventional photocatalysts, including titanium dioxide, can efficiently operate under near-ultraviolet (UV) irradiation, thanks to their wide band gap (E_g_ is equal to ~3.2 eV for bulk TiO_2_ [22]). In the case of the sunlight, for example, such materials can harvest less than 5% of its irradiation. Hence, it remains a challenge of primary importance to drive a photoresponse of disinfecting materials to visible light. More recently, several research groups have showed the destruction of microorganisms with nanomaterials based on plasmonic nanoparticles, which can efficiently generate heat, causing temperature-induced denaturation of proteins in pathogens [23]. Thus, the combination of photoactive and plasmonic nanoparticles, exhibiting antimicrobial properties when exposed to the light, can serve as an advanced modification for the filter made of a porous polymer membrane.

In this study, we propose a unique composition of the filter, i.e., membranes composed of porous polymer modified with photocatalytic and plasmonic nanostructures that ensure self-cleaning under excitation of easily accessible light sources. The filters are fabricated with the widely available Dr. Blade and spraying techniques. Their self-sterilization property is tested via an investigation of permeability to bacteria suspensions, solutions with solid state nanoparticles, and organic contaminants in the air flow, the presence/absence of which in the gaseous medium is determined by surface-enhanced Raman scattering (SERS) spectroscopy well-known for its excellent sensitivity due to the exploitation of the SERS-active substrates possessing plasmonic properties [24,25].

## 2. Materials and Methods

### 2.1. Materials

SU-8 2015 (MicroChem, Newton, MA, USA) and polymethylmethacrylate (PMMA) sheets of 2 mm thickness and 50 × 50 mm size (Plexiglas XT 20,070 MW approx. 155 kDa, Podolsk, Russia) were used to prepare polymers blend. Polylactic (PLA) and polyethylene terephthalate glycol (PET-G) were used for 3D printing of components utilized in the developed systems for both forming the polymers film and testing the porous polymer membrane permeability. Toluene (ACS reagent, ≥99.5%), acetone (ACS reagent, ≥99.5%), titanium (IV) isopropoxide (TTIP, ≥99.999%), hydrochloric acid (HCl, ACS reagent, 37%), isopropyl alcohol (IPA, 70% in H_2_O), methylene blue (MB), silver nitrate (AgNO_3_, ACS reagent, ≥99.0%), sodium citrate (ACS reagent, ≥99.0%) and sodium chloride (NaCl, ACS reagent, ≥99.0%) were supplied by Sigma Aldrich and used without additional purification. Agar American Type (Merck, Darmstadt, Germany) and LB Broth (VWR International LLC., Radnor, PA, USA) were used to prepare a medium for the bacterial growth according to protocols provided by the manufacturers. *Bacillus subtilis* Kwik-Stik 2 pack was purchased from Microbiologics and cultured following the supplier’s protocol. Water was purified with Milli-Q system (Millipore, Bedford, MA, USA).

### 2.2. Fabrication of Porous Polymer Membrane Modified with TiO_2_ and Ag Nanoparticles

#### 2.2.1. Formation and Porosification of Polymer Membrane

Prior to the polymer blend preparation, diced PMMA sheets (17.34 g) were dissolved in 100 mL of toluene heated up to 50 °C for 2 h. A PMMA-containing media (2 g) was added to SU-8 (4 g) and subjected to a constant stirring at 3500 rpm for 1 h using Multi Speed Vortex MSV-3500 (Biosan, Riga, Latvia). Next, a viscous polymer blend (1 mL) was dropped on the edge of a glass slide of 76 × 26 × 1 mm size fixed on the table of a hand-made Dr. Blade’s machine. The polymers drop was squeezed out to form a strip 26 mm in length to be equal to the glass slide width. Figure S1a shows the machine utilized for the polymer membrane formation equipped with stepper motors driven from a computer by a controller Arduino Uno to provide 2-axis (YZ) movement of the blade above the stationary table. The *Z*-axis position of the blade under the glass slide on the fixed table was set to produce ~20-µm-thick polymer film. Next, the polymer-coated glass slide was hardened on the hot plate at 95 °C for 10 min, followed by a photopolymerization step under the VL-6.LC 365/254 Vilber lamp (Vilber, France) with exposure at 365 nm wavelength for 1 min and final annealing on the hot plate at 95 °C for 5 min. The porosification of the resulting coating was achieved by the sample exposure to the 254-nm light of the Vilber lamp for 30 min to ensure photodegradation of the PMMA grains. Consequently, photodegraded PMMA grains were dissolved in acetone at 50 °C for 20 min. Finally, the porous SU-8 membrane was carefully repealed from the glass slide, thoroughly rinsed with water, and dried in a closed fume hood at 21 °C for 2 h.

#### 2.2.2. Fabrication of Titanium Dioxide Nanoparticles

TiO_2_ nanoparticles were formed by hydrothermal and sol-gel methods according to the slightly modified procedures described elsewhere [26,27]. Briefly, for the hydrothermal synthesis, a mixture of TTIP (0.56 mL), HCl (15 mL) and H_2_O (14.44 mL) was subjected to magnetic stirring for 5 min. Then, the resulting solution (30 mL) was poured into a stainless-steel container of a 50 mL internal volume for a further autoclaving at 150 °C for 2 h 45 min. Next, the solution was transferred from the autoclave to a ceramic crucible, evaporated at 90 °C for 1 h and annealed at 500 °C for 30 min. The sol-gel procedure started with stirring of the IPA (25 mL) and TTIP (3.75 mL) components for 5 min, followed by the addition of 3.5 mL H_2_O and one more stirring for 2 h. The resulting solution was aged at 21 °C for 24 h to produce gel. Finally, the gel was separated from the liquid phase, dried at 80 °C for 24 h, and annealed at 500 °C for 2 h.

#### 2.2.3. Colloidal Synthesis of the Ag Nanoparticles

Ag colloids were fabricated by a previously reported method [28] with minor modifications. Briefly, the solution containing 100 mL of water, 150 µL (for Ag nanoparticles of 60 nm diameter) or 210 (for Ag nanoparticles of 100 nm diameter) of 1 M AgNO_3_ silver ions was first heated to approximately 100 °C until boiling. Next, 100 µL of 1 M sodium citrate was added slowly while vigorously stirring the solution. The solution was kept at elevated temperatures until the color transformed to dark brown. Ag colloids were separated from a liquid phase by centrifugation at 6000 rpm for 15 min. We formed Ag colloids of two types: (i) 60 nm in a diameter as a plasmonic component in the membrane and (ii) 100 nm for utilizing instead of pathogens with the same size during the membrane permeability study.

#### 2.2.4. Combination of the Porous Polymer Membrane, TiO_2_ and Ag Nanoparticles

TiO_2_ and Ag nanoparticles were immobilized on the top side of the polymer membrane (i.e., on the external surface of the filter) by spraying from the IPA-based solution. Specifically, TiO_2_ (500 µg) and Ag (100 µg) nanoparticles were mixed with IPA (15 mL) in a plastic vial, which was then screwed on with a spraying nozzle. The outer opening of the nozzle was 0.5 × 0.5 mm size and 45 ° chamfers. The nozzle was fixed at approximately 12–13 mm distance from the polymer membrane to cover its surface shaped to a square with a 25 mm side. Therefore, three spray injections were made with a successive nozzle shift by 25 mm to form a coating on the entire top surface of the membrane inheriting the glass slide size. The 15 mL vial filled with the spraying mixture provided treatment of 50 membranes.

A prototype of the filter was made by the formation of a porous polymer membrane on the 2nd layer (filter pad) of the 3-layered facial medical mask fixed on the glass slide followed by spray-coating with the TiO_2_ and Ag nanoparticles solution.

#### 2.2.5. Bacteria Culturing and Suspension Preparation

A medium for the bacteria culturing was prepared by mixing agar (22.5 mg), LB Broth (22.5 g), and H_2_O (150 mL) followed by maintenance in a 2540EKA tabletop autoclave (Tuttnauer, Breda, The Netherlands) at 120 °C for 25 min. Then, the viscous solution was poured to the sterile Petri dishes and aged until gelation in the closed fume hood at 21 °C for 15–20 min. The bacteria spores were carefully inoculated on the gel surface using a cotton swab from the Kwik-Stik pack and kept in a Hettcube 600 incubator (Andreas Hettich GmbH & Co., Tuttlingen, Germany) at 36 °C for 72 h. The *bacillus subtilis* suspension was prepared by mixing 40 colonies of 1 mm diameter and saline (0.9 wt.% NaCl in H_2_O).

### 2.3. Characteriazation of the Porous Polymer Membrane Modified with TiO_2_ and Ag Nanoparticles

#### 2.3.1. Morphology Characterization

A porosity of the polymer membrane was revealed by the gravimetric method [29], measuring solid and porous SU-8 films of the same volume. A density of SU-8 was taken as 1.2 g/mL according to the Microchem guidelines.

The surface roughness of the polymer films before and after porosification was studied with atomic force microscope (AFM) NTEGRA Academia (NT-MTD, Zelenograd, Russia). The morphology of the polymer membranes, TiO_2_, and Ag nanoparticles was studied with scanning electron microscope (SEM) Hitachi S-4800 (Hitachi, Tokyo, Japan) equipped with a Bruker QUANTAX 200 energy dispersive X-ray spectrometer (EDXS) providing elemental composition analysis. TiO_2_ nanoparticles were additionally studied with 3D confocal Raman microscope-spectrometer Confotec NR500 (SOL Instruments, Minsk, Belarus) equipped with a 633-nm laser and X-ray diffractometer (XRD) Bruker D8 (Bruker, Billerica, MA, USA).

#### 2.3.2. Study of Photocatalytic Activity of TiO_2_ Nanoparticles and Thermal Properties of Ag Nanoparticles

We used MB as a model organic molecule to assess the ability of photocatalytic TiO_2_ and plasmonic Ag nanoparticles to destroy it because the MB solution is bleached if this organic dye is decomposed. This is a marker of photocatalytic activity and plasmonic heating of the nanoparticles. The decomposition efficiency (bleaching) of a 10^−5^ M MB water solution facilitated by the photocatalytic activity of the TiO_2_ nanoparticles and heating of the Ag nanoparticles upon light exposure was studied to select the optimal samples suitable for the polymer membrane modification. Four sets of 1.5 mL Eppendorf tubes filled with the 10^−5^ M MB solution were prepared: (1) pure MB solution as a control sample; MB solution with 100 µg TiO_2_ nanoparticles formed by (2) hydrothermal, (3) sol-gel method, and (4) with 100–300 µg Ag nanoparticles. The VL-6.LC 365/254 Vilber lamp (Vilber, Collégien, France) set to 365 nm wavelength was used to treat the control MB solution and MB/TiO_2_ solutions for 10 min. A LED projector Elektrostandard Elementary 033 FL (Elektrostandard, Moscow, Russia) with a power density set to 1.5 W was used to irradiate the control MB solution and Ag/MB solutions for 10 min period. Control samples from each series were not subjected to the light excitation but kept in a dark place for the same period of time as the treated solutions. The decomposition efficiency was estimated by comparison of the solutions absorption coefficient at the 664-nm wavelength, which corresponds to the maximal MB absorption band. The absorption coefficient was measured with spectrophotometer Ekros PE-5400 (Ekros, St. Petersburg, Russia), operating in visible and infrared ranges. This apparatus has a single-beam scheme and does not provide automatic registration of the complete absorption spectrum but allows to find out an absorption intensity at specific wavelength (664 nm in our case). That is why we presented the table with the absorption intensities and their processing for objective estimation of the MB decomposition. Thermal characterization of Ag nanoparticles upon LED exposure was performed using the Thermal imager Testo 871 (Testo SE&Co., Baden-Württemberg, Germany). Specifically, 100–300 µg Ag nanoparticles were mixed with 0.25 mL epoxy resin transparent for the visible light. This mixture was then placed over 20 × 20 mm^2^ area of the glass slide, producing thin film. This film was hardened in air at 21 °C for 30 min, following by its repulsion from a glass. A film of epoxy resin free of Ag nanoparticles was prepared as a control sample. Heating temperature was measured before and after film exposure with the LED projector for 10 min.

#### 2.3.3. Study of the Membrane Permeability and Disinfecting Effectiveness

The permeability of the developed membrane, filter prototype, and ordinary facial mask filter to the 100-nm Ag nanoparticles and bacteria solutions were studied using a system shown in Figure S1b. It composed of a holder for two glass vessels divided by a disks’ clamp with a circle opening in the center for the membrane fixing. The 100-nm nanoparticles or bacteria suspensions placed in the top vessel were dropped on the membrane clamped between the plastic disks and then were collected in the bottom vessel. The disinfection effectiveness of the samples containing bacteria was studied after the light exposure treatment. Specifically, the filtering sample was removed from the clamp and subjected to the LED projector or sunlight exposure for 10 min. The UV lamp was not used since the sunlight spectra overlaps the UV region. The experiments with the sunlight were performed at noon on a cloudless day. After drying the top and bottom side of the filtering samples with a wash swab, they were transferred to the surface of the agar Petri dishes. All Petri dishes were kept in a thermostat at 36 °C for 72 h and studied to reveal presence and number of the bacteria colonies.

The disinfection effectiveness of the porous polymer membrane and filter prototype spray-coated with TiO_2_ and Ag nanoparticles was studied using an airflow system depicted in Figure S1c and compared with the ordinary facial mask filter. This system contained a glass tube 25 mm in diameter with holders for the membrane or mask filter samples and a SERS-active substrate. The tube was connected to the vacuum pump VP115 (air volume displacement—51 L/min) to pull the air through the filter to the SERS-active substrate, which acted as a sensor of the contaminants in the flow. The sensing components were SERS-active substrates based on porous silicon and nanostructured silver coating as described elsewhere [30]. During these experiments, the pump pulled the air for 10 min under sunlight exposure. Subsequently, the SERS-active substrates were studied with a 3D confocal Raman microscope-spectrometer Confotec Duo (SOL Instruments, Minsk, Belarus) equipped with a 532-nm laser. All SERS measurements were made using ×100 objective (NA 0.95).

## 3. Results and Discussion

### 3.1. Morphology of the Polymer Membrane

**Figure 1:**
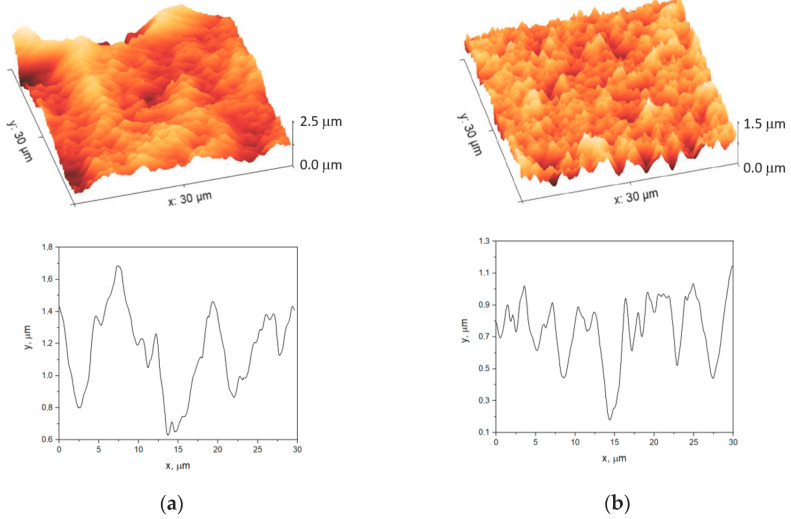
AFM images and profiles of (**a**) the SU-8 and PMMA membrane subjected to the thermal and photoinduced hardening and (**b**) the SU-8 membrane after removal of PMMA.

Figure 1 depicts AFM 3D topography images and corresponding cross section profiles of the hardened SU-8/PMMA membrane and that after the PMMA removal. It is seen from Figure 1a that the surface of PMMA-containing sample represents ridges of a 5–7.5-µm width and bumps of smaller sizes (1.0–4.5 µm in diameter). The most prominent surface features have ~1.1 µm height. The AFM image of the sample after PMMA photodegrading and etching shows absence of the wide ridges, but it is still enriched with the small bumps. The height of the surface features is lower and varies around 0.8 µm. Therefore, we can attribute the ridges presented in Figure 1a to the PMMA, which produces higher roughness compared to elements of the SU-8 skeleton in the form of the small bumps. Despite the surface morphology differences revealed, both membranes had nearly equal thickness of ~20 µm. The gravimetric analysis revealed the 30 ± 5% porosity of the SU-8/PMMA blend.

**Figure 2:**
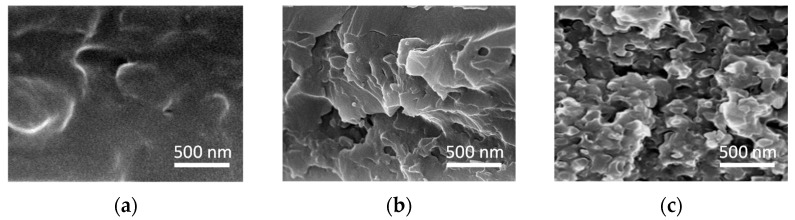
SEM top and cross section images of (**a**) the SU-8/PMMA membrane subjected to thermal and photoinduced hardening, (**b**) the SU-8/PMMA membrane after the PMMA photodegradation and (**c**) the SU-8 membrane after the PMMA removal.

The SEM top and cross section images of the membrane samples depicted in Figure 2 show that the solid structure of the SU-8/PMMA membrane (Figure 2a) turned into a loosened one (Figure 2b), thanks to a PMMA granulation. It is also seen from Figure 2c that the etching of a membrane in acetone produces porous SU-8 due to the dissolution of the PMMA granules. This SU-8 membrane exhibits a spongy morphology made of pores with approximately 50–300-nm diameters as seen, as shown in Figure 2c.

### 3.2. Characterization of Photocatalytical and Plasmonic Nanoparticles

#### 3.2.1. Morphology of Titanium Dioxide Nanoparticles

**Figure 3:**
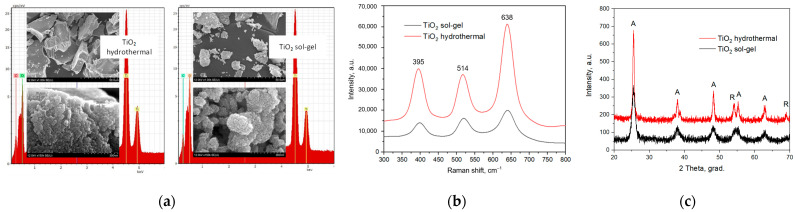
Results of structural characterization of TiO_2_ nanoparticles: (**a**) SEM images and EDX spectra, (**b**) Raman spectra and (**c**) XRD patterns (A—anatase, R—rutile).

Figure 3 depicts the structural characterization of TiO_2_ nanoparticles fabricated by the hydrothermal method. SEM images in Figure 3a indicate that the TiO_2_ nanoparticles form microscaled plates or aggregates. The EDX-spectra prove the presence of titanium and oxygen atoms in the samples. The TiO_2_ nanoparticles are spheres 22–28-nm in diameter for the hydrothermal samples and 17–23 nm for the sol-gel ones. The sol-gel samples are characterized by a more developed morphology and visually higher porosity, which is supposed to provide a larger specific surface area. The Raman spectra contain bands at 395, 514 and 638 cm^−1^ that characterize an anatase phase. The volume of the samples for the Raman study was the same for both cases but considering the higher porosity of the sol-gel TiO_2_ its Raman spectrum is three times less than that of the hydrothermal one. The XRD patterns demonstrate the dominance of the anatase phase for fabricated TiO_2_ nanoparticles over the rutile one.

#### 3.2.2. Morphology, Optical and Thermal Properties of Silver Nanoparticles

**Figure 4:**
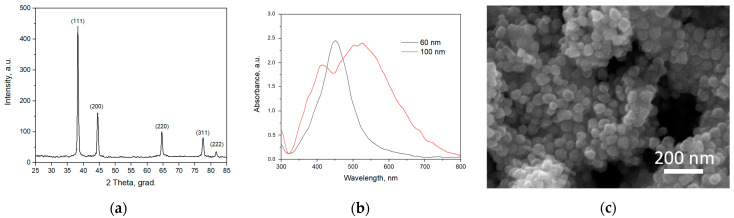
The results of structural and optical characterization of the Ag nanoparticles: (**a**) the XRD pattern, (**b**) the absorption spectra and (**c**) the SEM image.

The XRD pattern for the fabricated Ag nanoparticles indicates their polycrystalline nature (Figure 4a), while the SEM image Figure 4c suggests their quasi-spherical shape and mean diameter of 60 nm (Figure 4c). Two optical absorption spectra for 60 nm and 100 nm Ag nanoparticles shown in Figure 4b clearly indicate the surface plasmon resonance bands with maxima in a blue-green region of the visible light, which is consistent with other studies [31]. The fabricated 60-nm Ag nanoparticles presented in Figure 4c are thus assumed to provide effective decomposition of MB molecules and deactivation of bacteria governed by plasmonic heating under visible light excitation. Besides, we fabricated 100 nm Ag nanoparticles to utilize them as objects of size typical for viruses for polymer membrane permeability investigation.

**Figure 5:**
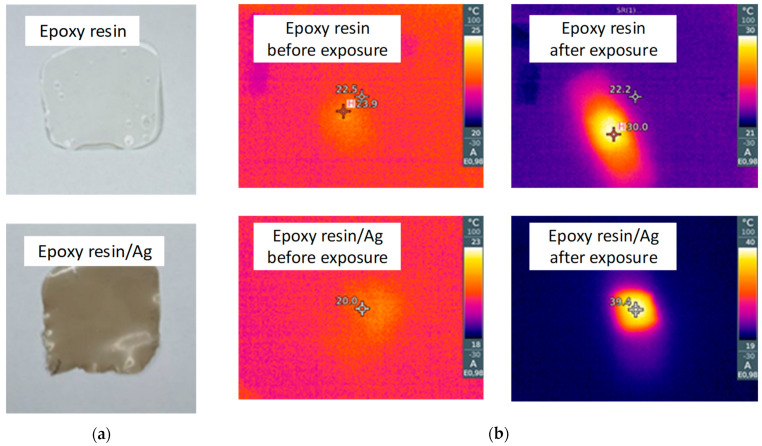
The study of the Ag nanoparticles thermal properties: (**a**) the photographs of the Ag-free and Ag-containing samples of the epoxy resin, (**b**) the corresponding thermal images of the samples after the LED projector exposure for 30 min.

Figure 5 depicts the results of the thermal measurements for the 60 nm Ag nanoparticles subjected to the LED projector exposure for 30 min. We performed the measurements with IR camera for the pure and covered with Ag nanoparticles epoxy resin film to find out if there is a heating facilitated by the plasmonic effect. As can be seen in Figure 5, the initial temperatures for both samples lie within the range of 19–21 °C, corresponding to that of the outer environment. On the other hand, the LED exposure of the samples produces temperature increases up to 40 °C for the sample with Ag nanoparticles, while the temperature for Ag-free sample increases only until 31 °C. These results indicate that the fabricated Ag nanoparticles are capable to produce a sufficient amount of heat to cause a microorganism’s deactivation, as consistent with literature data [23].

#### 3.2.3. Decomposition of Organic Dye Molecules

Optical absorption measurements for the pure and mixed with TiO_2_ or Ag nanoparticles 10^−5^ M MB solutions performed after UV or LED exposure suggested the samples with the highest decomposition effectiveness. The results of these measurements are collected in Table 1. Apparently, the control samples kept in the dark place for 10 min do not show absorption change. However, the absolute absorption coefficient of the MB solution is increased after addition of the TiO_2_ or Ag nanoparticles. This is because the nanoparticles facilitated the diffuse light scattering upon measurements, while the utilized instrument is only capable of the light detection passing normally through the sample. Moreover, it does not permit utilization of the reference sample to account for this signal loss, working only with the single-beam scheme. Nevertheless, we neglected this effect when assessing the efficiency of the MB decomposition by calculating the delta (in %) between the absorption intensities before and after UV or LED exposure. This approximation is possible in our case since neither TiO_2_ nor Ag nanoparticles absorbs light at a wavelength of 664 nm [31,32]. The TiO_2_-enriched MB solutions showed the most prominent LED-induced decrease in the absorption intensity by 43% and 47% for the hydrothermal and sol-gel samples, respectively. The lower photocatalytic activity of the hydrothermal TiO_2_ nanoparticles may be explained by their denser packaging and thus lower specific surface area per mass unit. The Ag nanoparticles provided the maximal absorption decrease at their minimal concentration. This is because Ag nanoparticles at high concentrations can aggregate prominently, leading to the deterioration of the plasmonic properties, and thus to less heat generation.

**Figure 6:**
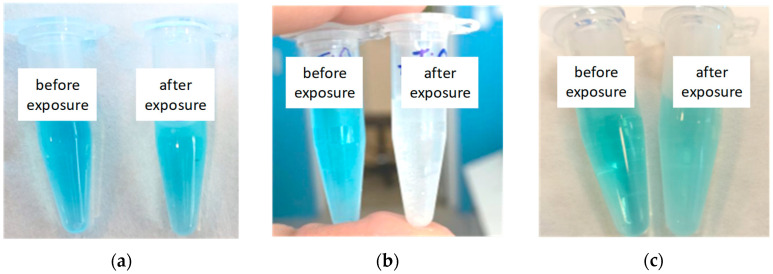
The Eppendorf tubes filled with the 10_−5_ M MB solutions (**a**) free of the TiO_2_ and Ag nanoparticles and containing (**b**) TiO_2_ or (**c**) Ag nanoparticles before and after UV or LED exposure.

Figure 6 shows digital photographs of the MB solutions mixed with TiO_2_ and Ag nanoparticles, which provided the most prominent decomposition of organic molecules. The sol-gel TiO_2_ nanoparticles in combination with the UV exposure were able to completely bleach the dye solution. The Ag nanoparticles (100 µg) together with the LED treatment also enabled a discoloration of the MB solution to a greater extent than just sole light exposure. Namely, the TiO_2_ and Ag nanoparticle samples were selected for further modification of the polymer membrane by the spraying procedure described above. An example of the EDX spectrum and SEM image of the membrane after spraying can be found in Figure S2.

### 3.3. Study of Disinfecting Effectiveness

Experiments on the permeability of the TiO_2_/Ag/polymer membrane to the Ag nanoparticles 100 nm in diameter suggest that, in contrast to the pure water, the silver-containing aqueous solution cannot pass through the system (see Figure S3). The bottom side of the membrane remains dry, indicating that the pores are most probably clogged with the nanoparticles from the solution. Therefore, we may assume that the pathogens with sizes over 100 nm travelling on the water drops in a moist air and adsorbing on the top side of the membrane will not penetrate to its bottom side.

**Figure 7:**
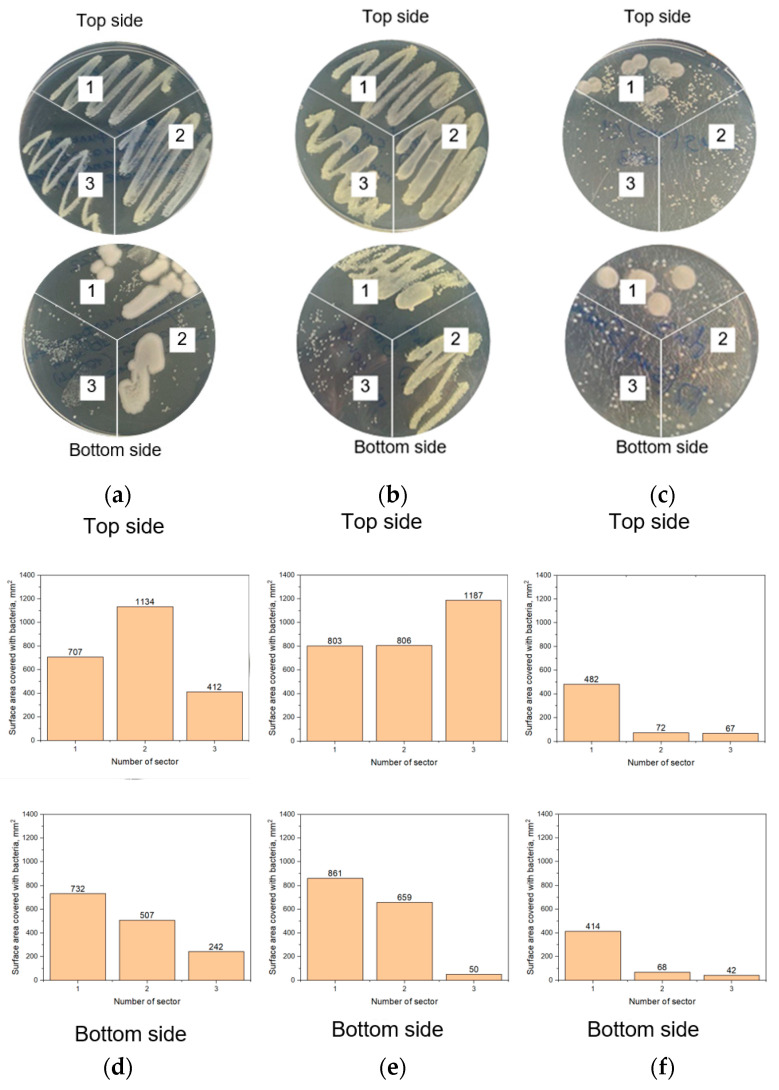
The top views of the Petri dishes filled with agar gel and cultured bacteria wash-swabbed from the filtering materials subjected to dropping the bacterial suspension on the top side (**a**) before the light exposure, (**b**) after the LED projector exposure for 30 min and (**c**) after the sunlight exposure for 30 min. Sector #1—the medical face mask filter, sector #2—the prototype made of TiO_2_/Ag/polymer membrane on the medical face mask filter, sector #3—the TiO_2_/Ag/polymer membrane: (**d**–**f**)—histograms of surface area of the Petri dish sectors covered with bacteria, which correspond to (**a**–**c**) photos.

Figure 7 illustrates the experimental results on the light-facilitated disinfection of the middle filter of the standard 3-layered medical facial mask. For these experiments, three samples were studied: (1) the standard the medical face mask filter, (2) the prototype made of TiO_2_/Ag/polymer membrane on the medical face mask filter, and (3) the TiO_2_/Ag/polymer membrane. To study the sterilization properties of all these samples, wash swabs were taken from their top and bottom sides after the bacteria permeability test, following by the LED or sun exposure. The bacteria collected in this way were cultured from the wash swabs on the agar gel surface in sectors 1–3 of the Petri dishes. We defined the bacteria-covered area of each sector in the Petri dishes using ImageJ software to perform a kind of quantitative assessment of the bacteria amount since counting a number of the bacteria colonies was impossible for some sectors. Figure 7 confirms that the bacteria remained on both sides of samples #1 and #2. Therefore, inserting the filter from the standard mask into the prototype is undesirable since it promotes penetrating bacteria spores to the bottom side. In contrast, the results in Figure 3c clearly show much fewer bacteria colonies on the top side and practically no colonies on the bottom side of the TiO_2_/Ag/polymer membrane after LED excitation. More importantly, the sun-exposed sample of the TiO_2_/Ag/polymer membrane exhibits a negligible amount of the bacteria colonies on both sides. Nevertheless, we should accept a negative penetration effect of the microbes’ species through the developed membrane. The barrier effect preventing penetration of the solid inorganic nanostructures observed in the course of the permeability experiments upon 100-nm Ag nanoparticle utilization can be ascribed to the poor wettability of the top membrane surface and thus deserves additional study.

**Figure 8:**
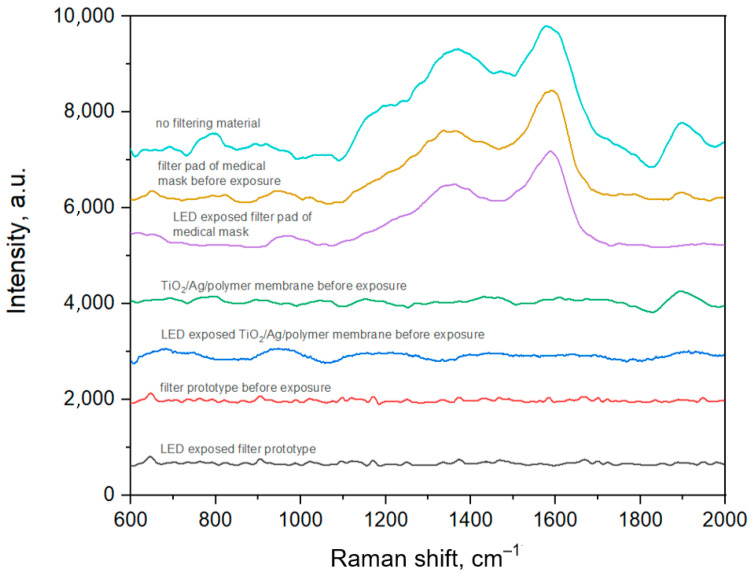
The results of SERS-analysis of air pulled out through the filtering materials before and after the LED exposure: average SERS-spectra for the medical mask filter pad, prototype and TiO_2_/Ag/polymer membrane are depicted.

The flow experiments performed with samples 2 (prototype) and 3 (TiO_2_/Ag/polymer membrane) indicate that they are capable of cleaning the inhaled air from organic contaminants. As can be seen from Figure 8, the Raman spectra recorded on the SERS-active substrates installed behind the standard mask filter are typical for the amorphous carbon signature (1366 and 1574 cm^−1^) that appears when organic molecules are burned due to heating induced by the laser and plasmonic nanoparticles. It should be noted that the mask filter pad did not provide air cleaning upon excitation with the sunlight as also compared during collecting the SERS-spectra array shown in Figure S4. 

On the other hand, both TiO_2_/Ag/polymer membrane and the corresponding face mask prototype provided the air cleaning as follows from the background spectra collected with the SERS-active substrates, which do not contain any characteristic Raman bands of the organic contaminants.

## 4. Conclusions

In this study, we developed a filtering material for facial masks, which is capable of the trapping and subsequent inactivation of bacteria under white LED or sunlight irradiation. Such a functionality was achieved via the modification of a composite membrane based on porous polymer (SU-8) with photocatalytic (TiO_2_) and plasmonic (Ag) nanoparticles. The porous polymer is produced by means of a computer numerical control machine, which rolls the SU-8/PMMA blend into a ~20-µm-thick membrane followed by its thermal/UV hardening and porosification via the 254-nm photodegradation and acetone etching of PMMA. The resulting SU-8 membrane exhibits a spongy morphology made of pores approximately 50–300 nm in diameter. TiO_2_ nanoparticles were prepared by hydrothermal and sol-gel techniques. Colloidal synthesis was utilized to fabricate Ag nanoparticles. The TiO_2_ photocatalytic activity under UV excitation as well as a photothermal effect generated by plasmonic Ag nanoparticles subjected to LED irradiation were studied by the assessment of MB decomposition. The sol-gel TiO_2_ nanoparticles in combination with the UV exposure were able to completely bleach the dye solution. The Ag nanoparticles (100 µg) together with the LED treatment also enabled the discoloration of the MB solution to a greater extent than solely light exposure. The TiO_2_ and Ag nanoparticles samples were selected for further modification of the polymer membrane by the spraying procedure. We demonstrated that, in contrast to the filter of the standard facial medical mask, the polymer membrane modified with spray-coated TiO_2_ and Ag nanoparticles prevents the penetration of *bacillus subtilis* from its top to bottom side and significantly inhibits bacterial growth when exposed to LED or sunlight. Since the developed filter was able to move air at 51 L per min during testing for 10 min, we conclude that it can provide free breathing. Such a conclusion is supported by data on tidal volume, i.e., the volume of air moving in or out of the lungs in one breathing cycle, equal to 7 mL of breathing air per 1 kg body weight in one breath for adult [33]. This means that a 70-kg adult breathes ~500 mL of air per breath while a child weighing 10 kg has a tidal volume of 70 mL and breathes 2.1 L of air per min [34]. Therefore, the designed and engineered composite membrane is argued to be a prospective model for the further development of self-disinfecting filters, the application area of which can be extended to small scale photosynthesis as well as water and air cleaning.

## Figures and Tables

**Table 1:** Mean absorption band intensity (a.u.) of the MB solutions free of and containing TiO_2_ and Ag nanoparticles kept in the dark place or exposed to the UV lamp or LED projector irradiation for 10 min (three measurements were made for each sample).

Sample Type	Absorption, a.u.				Absorption Delta for Fresh and Exposed Samples, %
	Fresh	Kept in Dark Place	Exposed	Exposed and Corrected for Control MB	
MB	22	22	15 (UV)	-	32
MB + TiO_2_ (hydroth.)	88	86	43 (UV)	50	43
MB + TiO_2_ (sol-gel)	81	80	36 (UV)	43	47
MB	22	22	20 (LED)	-	9
MB + Ag (100 µg)	28	27	18 (LED)	20	29
MB + Ag (200 µg)	32	32	22 (LED)	24	25
MB + Ag (300 µg)	34	34	27 (LED)	29	15

## Data Availability

Not applicable.

## Supplementary Materials

The following supporting information can be downloaded at: https://www.mdpi.com/article/10.3390/polym15030726/s1, Figure S1: The systems for the formation and testing the experimental samples: (a) the Dr. Blade machine for formation of the polymer membrane, (b) the permeability testing system and (c) the airflow system.; Figure S2: EDX spectrum and SEM images of the polymer membrane after Ag/TiO2 spraying.; Figure S3: Experiments on the permeability of the TiO_2_/Ag/polymer membrane to the Ag nanoparticles with 100 nm diameters showing that in contrast to (a) pure water (b) the silver-containing aqueous solution is not able to leak out; the upper photos show the top of the clamped plastic disks with the filter and a drop of the (a) Ag-free or (b) Ag-containing solution on it; the corresponding bottoms of the disks are presented below demonstrating that the Ag-free solution went through the filter while the Ag-containing solution did not; Figure S4: The results of SERS-analysis of air pulled out through the filtering materials before and after the LED exposure: arrays of the spectra collected on the SERS-active substrates installed after (a) the medical mask filter and (**b**) the TiO_2_/Ag/polymer membrane; z axis shows the number of the SERS-spectra registered during the SERS-active substrate mapping.
