# Supplementary material for: Polymer Membrane Modified with Photocatalytic and Plasmonic Nanoparticles for Self-Cleaning Filters
Source: Polymers (Basel). 2023 Jan 31;15(3):726. doi: 10.3390/polym15030726 (PMC9920405; doi:10.3390/polym15030726)
Supplement: Supplementary file 1 [file polymers-15-00726-s001.zip › polymers-2132888-supplementary.pdf]

polymers

MDPI

Supplementary Information

Polymer Membrane Modified with Photocatalytic and Plasmonic Nanoparticles for Self-Cleaning Filters

Aliaksandr Burko [1,2], Siarhei Zavatski [1,2], Arina Baturova [1,2], Makhina Kholiboeva [1], Julia Kozina [1], Kseniya Kravtsunova [3], Vladimir Popov [1,2], Artem Gudok [1], Sergey Dubkov [2], Stanislav Khartov [4] and Hanna Bandarenka [1,2,*]

[1] Applied Plasmonic Laboratory, Belarusian State University of Informatics and Radioelectronics, 220013 Minsk, Belarus
[2] Institute of Advanced Materials and Technologies, National Research University of Electronic Technology, Moscow 124498, Russia
[3] Department of General and Applied Physics, Moscow Institute of Physics and Technology, Moscow 141701, Russia
[4] Federal Research Center «Krasnoyarsk Scientific Center», Siberian Branch, Russian Academy of Sciences, Moscow 119991, Russia
* Correspondence: h.bandarenka@bsuir.by

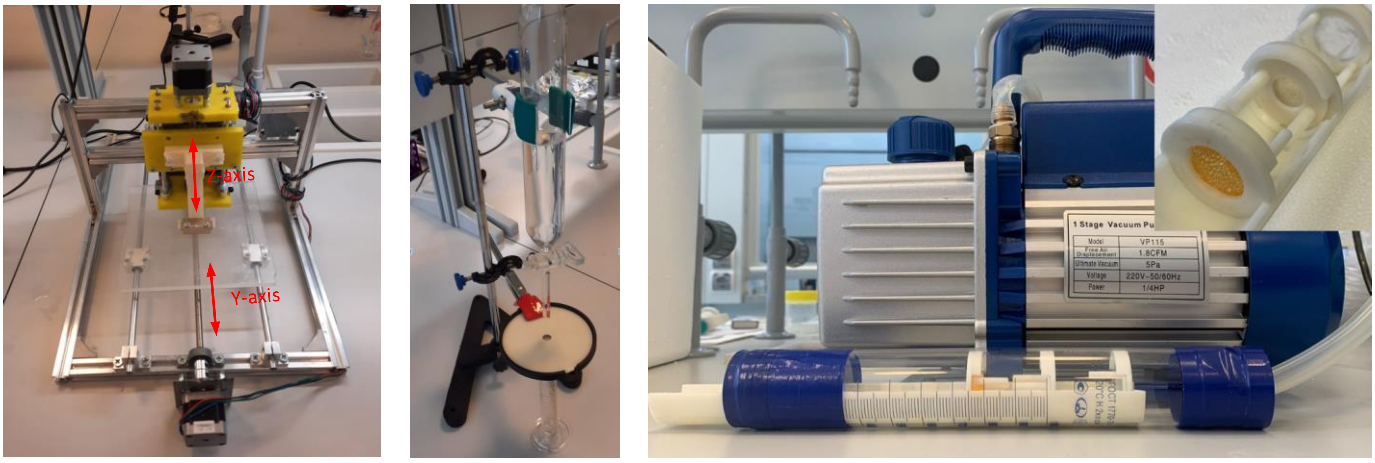

(**a**) (**b**) (**c**)

Figure S1. The systems for the formation and testing the experimental samples: (a) the Dr. Blade machine for formation of the polymer membrane, (b) the permeability testing system and (c) the airflow system.

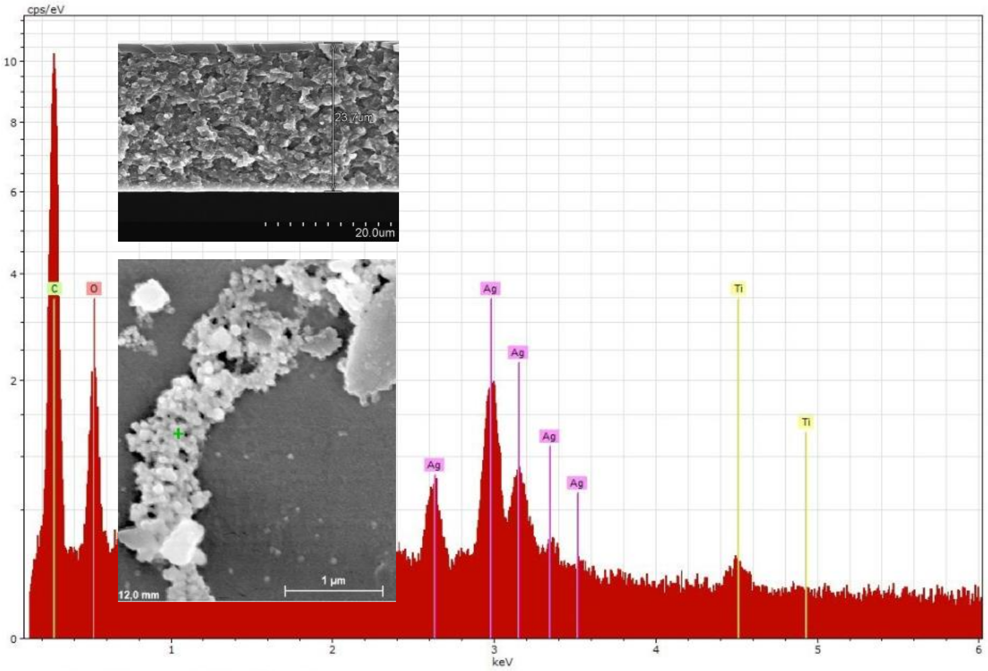

Figure S2. EDX spectrum and SEM images of the polymer membrane after Ag/TiO_2 spraying.

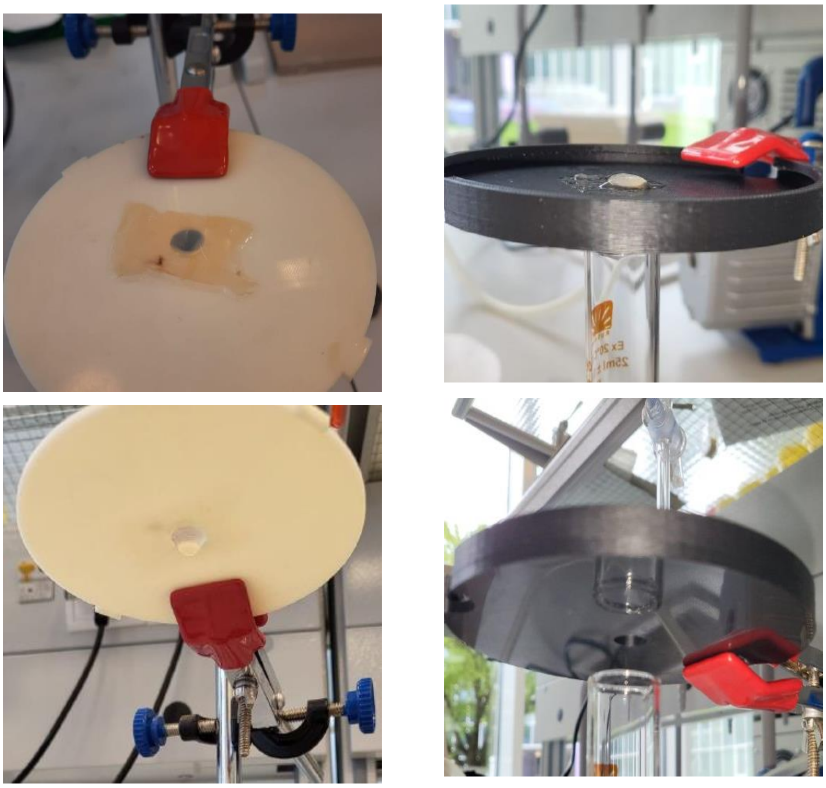

(**a**) (**b**)

Figure S3. Experiments on the permeability of the TiO_2/Ag/polymer membrane to the Ag particles with 100 nm diameters showing that in contrast to (**a**) pure water (**b**) the silver-containing aqueous solution is not able to leak out; the upper photos show the top of the clamped plastic disks with the filter and a drop of the (**a**) Ag-free or (**b**) Ag-containing solution on it; the corresponding bottoms of the disks are presented below demonstrating that the Ag-free solution went through the filter while the Ag-containing solution did not.

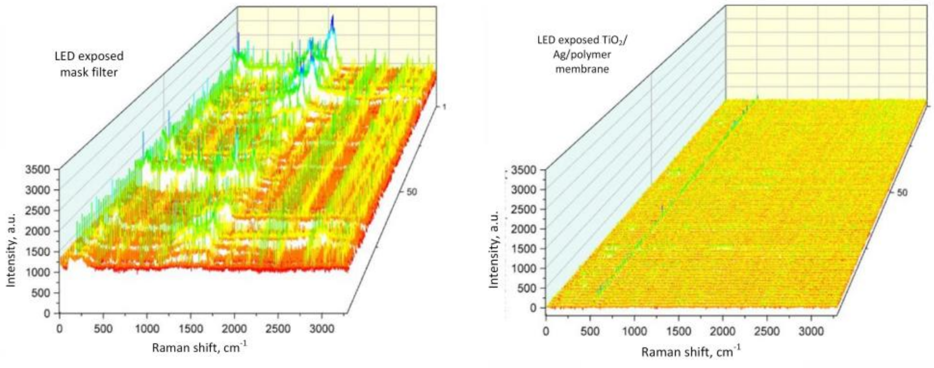

(**a**) (**b**)

Figure S4. The results of SERS-analysis of air pulled out through the filtering materials before and after the LED exposure: arrays of the spectra collected on the SERS-active substrates installed after (**a**) the medical mask filter and (**b**) the TiO_2/Ag/polymer membrane; z axis shows the number of the SERS-spectra registered during the SERS-active substrate mapping.
